# Selective Oxidation Using In Situ-Generated Hydrogen Peroxide

**DOI:** 10.1021/acs.accounts.3c00581

**Published:** 2023-12-20

**Authors:** Richard J. Lewis, Graham J. Hutchings

**Affiliations:** Max Planck−Cardiff Centre on the Fundamentals of Heterogeneous Catalysis FUNCAT, Cardiff Catalysis Institute, School of Chemistry, Cardiff University, Cardiff, CF24 4HQ, United Kingdom

## Abstract

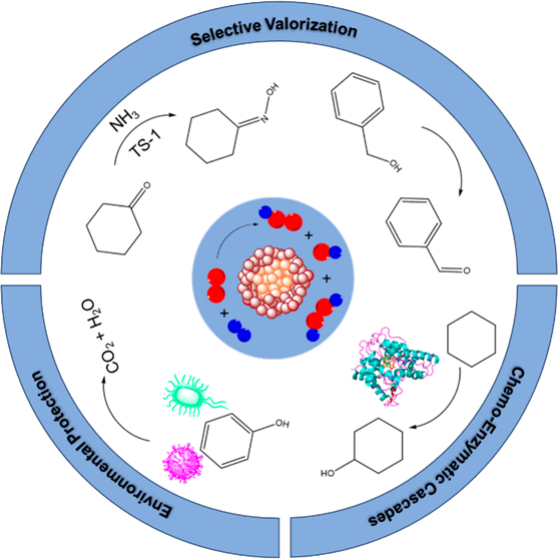

Hydrogen peroxide (H_2_O_2_) for industrial applications is manufactured through an indirect process that relies on the sequential reduction and reoxidation of quinone carriers. While highly effective, production is typically centralized and entails numerous energy-intensive concentration steps. Furthermore, the overhydrogenation of the quinone necessitates periodic replacement, leading to incomplete atom efficiency. These factors, in addition to the presence of propriety stabilizing agents and concerns associated with their separation from product streams, have driven interest in alternative technologies for chemical upgrading. The decoupling of oxidative transformations from commercially synthesized H_2_O_2_ may offer significant economic savings and a reduction in greenhouse gas emissions for several industrially relevant processes. Indeed, the production and utilization of the oxidant in situ, from the elements, would represent a positive step toward a more sustainable chemical synthesis sector, offering the potential for total atom efficiency, while avoiding the drawbacks associated with current industrial routes, which are inherently linked to commercial H_2_O_2_ production. Such interest is perhaps now more pertinent than ever given the rapidly improving viability of green hydrogen production.

The application of in situ-generated H_2_O_2_ has been a long-standing goal in feedstock valorization, with perhaps the most significant interest placed on propylene epoxidation. Until very recently a viable in situ alternative to current industrial oxidative processes has been lacking, with prior approaches typically hindered by low rates of conversion or poor selectivity toward desired products, often resulting from competitive hydrogenation reactions. Based on over 20 years of research, which has led to the development of catalysts for the direct synthesis of H_2_O_2_ that offer high synthesis rates and >99% H_2_ utilization, we have recently turned our attention to a range of oxidative transformations where H_2_O_2_ is generated and utilized in situ. Indeed, we have recently demonstrated that it is possible to rival state-of-the-art industrial processes through in situ H_2_O_2_ synthesis, establishing the potential for significant process intensification and considerable decarbonization of the chemical synthesis sector.

We have further established the potential of an in situ route to both bulk and fine chemical synthesis through a chemo-catalytic/enzymatic one-pot approach, where H_2_O_2_ is synthesized over heterogeneous surfaces and subsequently utilized by a class of unspecific peroxygenase enzymes for C–H bond functionalization. Strikingly, through careful control of the chemo-catalyst, it is possible to ensure that competitive, nonenzymatic pathways are inhibited while also avoiding the regiospecific and selectivity concerns associated with current energy-intensive industrial processes, with further cost savings associated with the operation of the chemo-enzymatic approach at near-ambient temperatures and pressures. Beyond traditional applications of chemo-catalysis, the efficacy of in situ-generated H_2_O_2_ (and associated oxygen-based radical species) for the remediation of environmental pollutants has also been a major interest of our laboratory, with such technology offering considerable improvements over conventional disinfection processes.

We hope that this Account, which highlights the key contributions of our laboratory to the field over recent years, demonstrates the chemistries that may be unlocked and improved upon via in situ H_2_O_2_ synthesis and it inspires broader interest from the scientific community.

## Key References

- FreakleyS. J.; KochiusS.; van MarwijkJ.; FennerC.; LewisR. J.; BaldeniusK.; MaraisS. S.; OppermanD. J.; HarrisonS. T. L.; AlcaldeM.; SmitM. S.; HutchingsG. J.A chemo-enzymatic oxidation cascade to activate C-H bonds with in situ generated H_2_O_2_. Nat. Commun.2019, 10( (1), ), 417810.1038/s41467-019-12120-w31519878 PMC6744418.^1^*The wide conditions gap that exists between chemo-catalysts and enzymes can be bridged, facilitating efficient C–H bond oxidation.*
- RichardsT.; HarrhyJ. H.; LewisR. J.; HoweA. G. R.; SuldeckiG. M.; FolliA.; MorganD. J.; DaviesT. E.; LoveridgeE. J.; CroleD. A.; EdwardsJ. K.; GaskinP.; KielyC. J.; HeQ.; MurphyD. M.; MaillardJ.; FreakleyS. J.; HutchingsG. J.A residue-free approach to water disinfection using catalytic in situ generation of reactive oxygen species. Nat. Catal.2021, 4( (7), ), 575–58510.1038/s41929-021-00642-w.^2^*AuPd alloy formation promotes the release of oxygen-based species generated as intermediates during H*_*2*_*O*_*2*_*direct synthesis, with these radicals offering high biocidal efficacy.*
- CrombieC. M.; LewisR. J.; TaylorR. L.; MorganD. J.; DaviesT. E.; FolliA.; MurphyD. M.; EdwardsJ. K.; QiJ.; JiangH.; KielyC. J.; LiuX.; Skjøth-RasmussenM. S.; HutchingsG. J.Enhanced Selective Oxidation of Benzyl Alcohol via In Situ H_2_O_2_ Production over Supported Pd-Based Catalysts. ACS Catal.2021, 11( (5), ), 2701–271410.1021/acscatal.0c04586.^3^*The in situ synthesis of H*_*2*_*O*_*2*_*over Pd coupled with Fenton’s chemistry-mediated radical generation offers improved reactivity in alcohol oxidation.*
- LewisR. J.; UeuraK.; LiuX.; FukutaY.; DaviesT. E.; MorganD. J.; ChenL.; QiJ.; SingletonJ.; EdwardsJ. K.; FreakleyS. J.; KielyC. J.; YamamotoY.; HutchingsG. J.Highly efficient catalytic production of oximes from ketones using in situ-generated H_2_O_2_. Science2022, 376( (6593), ), 615–62010.1126/science.abl482235511983 .^4^*In situ H*_*2*_*O*_*2*_*generation can rival current industrial routes to chemical upgrading and offer improved process efficiencies.*

## Introduction

The utilization of H_2_O_2_ for chemical synthesis typically offers exceptional selectivities, rivaling those achieved by organic peroxides and stoichiometric oxidants (e.g., perchlorate and permanganate) while also avoiding the large quantities of byproducts and subsequent purification costs associated with these conventional reagents. Additionally, H_2_O_2_-mediated processes typically allow for lower operating temperatures and improved selectivities compared to alternative aerobic pathways. Currently, the large-scale production of H_2_O_2_ is met almost entirely by the highly efficient anthraquinone oxidation (AO) process, where substituted anthraquinones are first hydrogenated and subsequently oxidized, regenerating the quinone carrier and producing an equimolar amount of the oxidant.

A long-standing goal of catalysis has been the synthesis of H_2_O_2_ directly from the elements, which would allow for decentralized production and significantly lower capital costs and emission release compared to traditional approaches. However, despite extensive study,^5−8^ including by our own laboratory^9,10^ a direct synthesis alternative to the current means of H_2_O_2_ production has not yet emerged. In recent years, a significant hindrance to a direct approach, namely, limited catalyst selectivity, has been overcome, with a growing selection of catalyst formulations reported that can achieve near total selectivity toward H_2_O_2_.^8−11^ Importantly, these works have demonstrated that high selective utilization of H_2_ may be achieved in the absence of the halide and acid stabilizers that had typically been necessary to inhibit competitive H_2_O_2_ degradation pathways, particularly over Pd-only catalysts.^12^ However, in order to rival the AO process it is necessary for concentrations of H_2_O_2_ of approximately 5 vol % to be obtained, minimizing costs associated with product separation, prior to shipping. To date, the production of such concentrations has been achieved only through the use of hydrogen/oxygen gas mixtures within the explosive regime, an approach which is clearly not practical.^13^ As with H_2_O_2_ generated ex situ by traditional industrial approaches, that synthesized via the direct combination of H_2_ and O_2_ for chemical synthesis would still likely require storage and the dilution of product streams through continual dosing of the oxidant.

Over the past decade, our laboratory has extensively investigated the use of in situ-generated H_2_O_2_ for a range of selective oxidative processes and identified the enhanced performance metrics that may be achieved compared to alternative approaches. In this Account, we seek to illustrate the versatility of such chemistry and promote wider interest in the development of novel, more sustainable technologies centered around the in situ production of H_2_O_2_. Beyond the realms of traditional heterogeneous catalysis, we further demonstrate that new frontiers in oxidative chemistry are yet to be fully realized, with a particular focus on the application of such technology in chemo/enzymatic cascades and in pollutant remediation.

## Challenging Current Industrial Processes

The most pertinent examples of industrial feedstock valorization reliant on preformed H_2_O_2_ are perhaps the epoxidation of propylene to propylene oxide (HPPO process) and the ammoximation of cyclohexanone, a key process in the production of the Nylon-6 monomer, ε-caprolactam. In both cases, the utilization of preformed H_2_O_2_ has presented significant improvements compared to alternative technologies, primarily associated with lower energy inputs and reduced purification costs. Further interest has been placed on the production of other bulk chemicals, including adipic acid, cyclohexanone, cyclohexanol, phenol, and methanol, with many processes reaching relatively advanced stages of development. However, progression to industrial production has been precluded, at least in part, by financial considerations, with the high cost of preformed H_2_O_2_ relative to that of the desired product often prohibiting commercialization. However, through effective in situ production of the oxidant, considerable cost reductions may be achieved, which when coupled with improved environmental credentials improves commercial viability.

Although widely investigated academically, here we raise special mention of the long-standing investigation by Haruta and co-workers,^14−16^ among many renowned laboratories,^17−22^ into the production of propylene oxide, and the application of in situ-generated H_2_O_2_ for feedstock valorization has faced a number of challenges. These include poor selective H_2_ utilization, rapid catalyst deactivation, and the formation of complex product mixtures, necessitating extensive purification and the inclusion of promoters. Indeed, in many cases, it is the presence of H_2_, required to generate H_2_O_2_ in situ, that largely promotes the formation of such byproducts.^20,23^ Such concerns are not limited to alkene epoxidation, with product distributions for a range of transformations influenced by competitive unselective hydrogenation pathways.^24,25^ It is the overcoming of these challenges that has motivated extensive research from our laboratory, with particular focus placed on the application of Pd-based catalysts for (i) alkane upgrading,^26−31^ (ii) alcohol oxidation,^3,32−34^ and recently (iii) the ammoximation of cyclohexanone (and other cyclic ketones) to the corresponding oxime.^4,35,36^ Regarding alkane oxidation, we direct the reader to our recent Account on methane valorization for an extensive discussion of our contribution to this field.^37^

## Ketone Ammoximation

The development of the titanosilicate TS-1 by EniChem can be considered to be one of the most important innovations in industrial heterogeneous catalysis in recent decades, offering exceptional activities and selectivities for the oxidative transformation of many small molecules, as dictated by the relatively limited pore size of TS-1 (∼5.5 Å), which consists of a 10-membered ring (MFI) framework. In particular, the industrial production of cyclohexanone oxime via the TS-1/H_2_O_2_ mediated ammoximation process represents a considerable improvement over conventional approaches, which generate large quantities of low-value byproducts.^38^ Unlike alternative H_2_O_2_-driven chemical transformations, which utilize the oxidant as a source of oxygen-based radicals the ammoximation mechanism is considered to rely on the diffusion of H_2_O_2_ to Ti^IV^ active sites within the TS-1 framework, forming a Ti-OOH moiety, that is utilized in the formation of hydroxylamine, which subsequently reacts noncatalytically with the ketone to generate the corresponding oxime.^39,40^ The TS-1/H_2_O_2_ approach is highly efficient, avoiding the production of considerable quantities of byproducts associated with conventional approaches that rely on hydroxylamine salts and indeed near total selectivity to the oxime may be achieved, with oxime yields in excess of 98% reported.^41^ However, given the reaction conditions utilized (high temperature and an elevated pH), an excess of H_2_O_2_ is typically utilized to account for the degradation of the oxidant. The inefficient use of H_2_O_2_ may be considered to be an additional source of process inefficiency and a challenge that applies to many preformed H_2_O_2_-mediated processes.^42^

**Figure 1:**
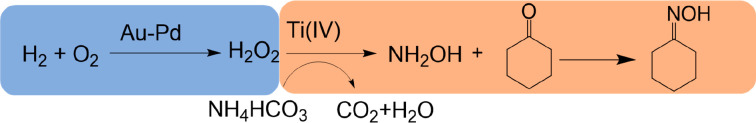
Simplified reaction scheme for the ammoximation of cyclohexanone via in situ H_2_O_2_ synthesis. Note that a wide conditions gap exists between the H_2_O_2_ direct synthesis and ketone ammoximation reactions, with the former favored by subambient temperatures and acidic conditions while the latter requires elevated reaction temperatures and basic conditions.

**Figure 2:**
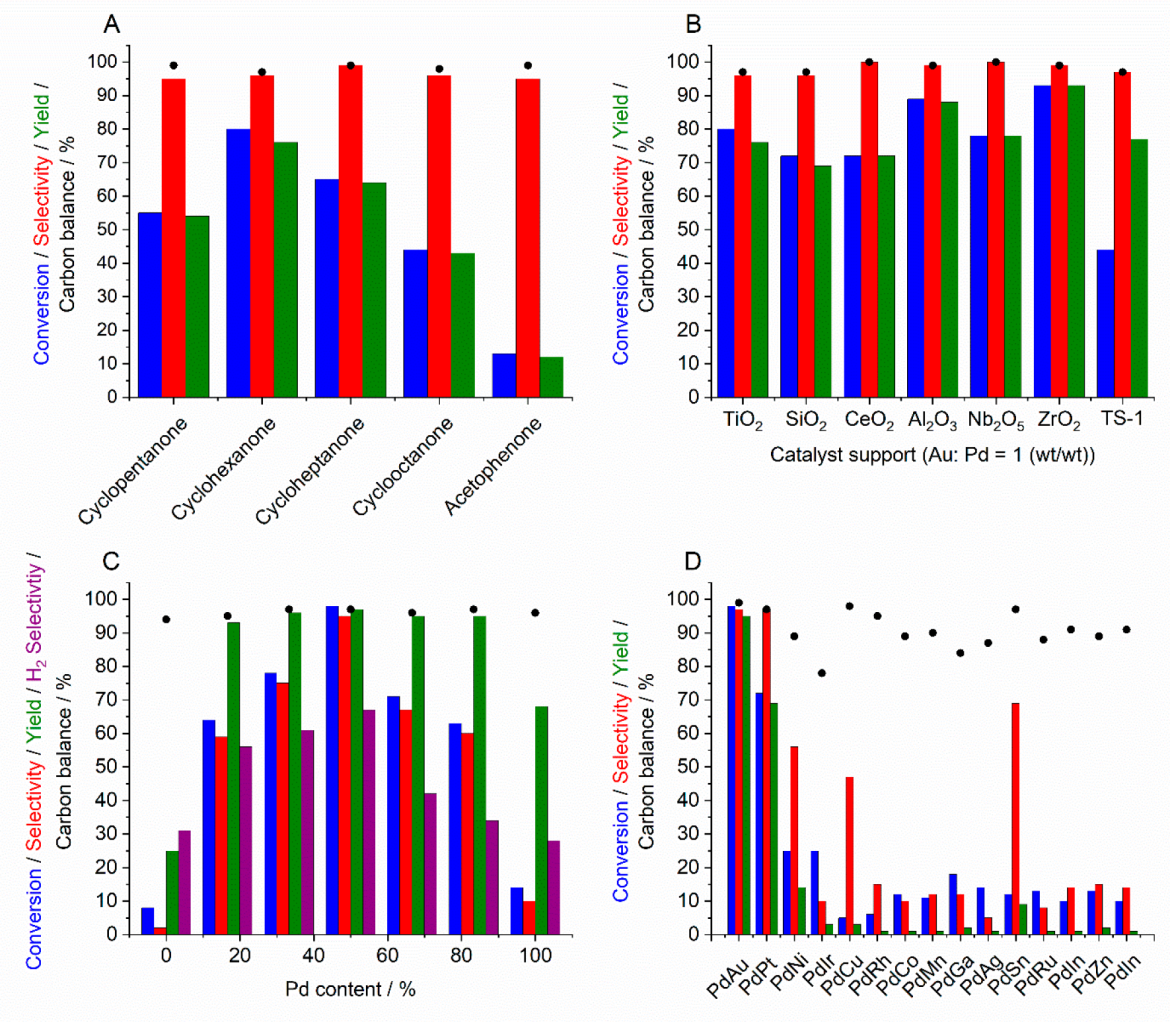
Ketone ammoximation via in situ H_2_O_2_ synthesis. (A) Catalytic activity of a 0.66% AuPd/TiO_2_ catalyst, used in conjunction with TS-1, toward the in situ ammoximation of a range of ketones. (B) Comparison of catalyst support on the activity of AuPd nanoalloys toward the in situ ammoximation of cyclohexanone. Note that with the exception of the TS-1 catalyst, all other formulations were used in conjunction with TS-1 (0.075 g). (C) Effect of the Au:Pd ratio on the catalytic activity of 0.66% PdAu/TS-1 catalysts toward the in situ ammoximation of cyclohexanone. (D) Catalytic activity of 0.66% PdX/TS-1 catalysts toward the in situ ammoximation of cyclohexanone. Note that Pd:X = 1:1 (w/w). Reaction conditions (A, B): Cyclohexanone (2 mmol), NH_4_HCO_3_ (4 mmol), 5% H_2_/N_2_ (420 psi), 25% O_2_/N_2_ (160 psi), catalyst (0.075 g), *t*-BuOH (5.9 g), H_2_O (7.5 g), reaction time 3 h, reaction temperature 80 °C, stirring speed 800 rpm. Reaction conditions (C, D): Same as above, but the reaction time is 6 h.

Significant process improvements may be achieved by decoupling the commercial ammoximation process from the industrial route to H_2_O_2_ production. However, until recently, alternative efforts focused on an integrated process have generated complex and potentially harmful product mixtures, necessitating energy-intensive distillation steps, which would preclude adoption at scale. In particular, the synthesis of H_2_O_2_ through the partial oxidation of isopropanol and subsequent utilization in ketone ammoximation has been described but yields considerable levels of byproducts (including acetone as well as phosphoric and acetic acids), in addition to residual isopropanol.^43^ Such complex product streams are unfavorable, requiring energy-intensive purification steps before application. Notably, the presence of H_2_O_2_ and acetone also poses a serious risk through the formation of shock explosives such as diacetone peroxide. By comparison, our one-pot approach to in situ ketone ammoximation avoids the concerns of alternative approaches and utilizes the ability of immobilized Pd-based nanoparticles to synthesize H_2_O_2_, which is reacted with ammonia by TS-1 to form a hydroxylamine intermediate, in a manner similar to the current industrial process (Figure 1), with the in situ route offering high selectivity for a range of cyclic oximes (Figure 2A).

Such systems, utilized either as a physical mixture of two separate catalysts or as a composite material that can catalyze both individual reaction pathways, have been demonstrated to offer exceptional reactivity and selectivity toward a range of oximes, with yields comparable to that achieved by the current state-of-the-art industrial process (Figure 2B). In particular, the formation of AuPd nanoalloys has been found to offer improved performance compared to Pd-only analogues^4^ or alternative Pd-based formulations (Figure 2C,D)^35^ despite the enhanced rate of H_2_O_2_ synthesis observed over monometallic Pd formulations, under conditions optimized for the stability of the oxidant. Such observations may be attributed to the improved stability of bimetallic AuPd species compared to that of monometallic Pd analogues and the increased selective utilization of H_2_ over alloyed surfaces.^4^

**Figure 3:**
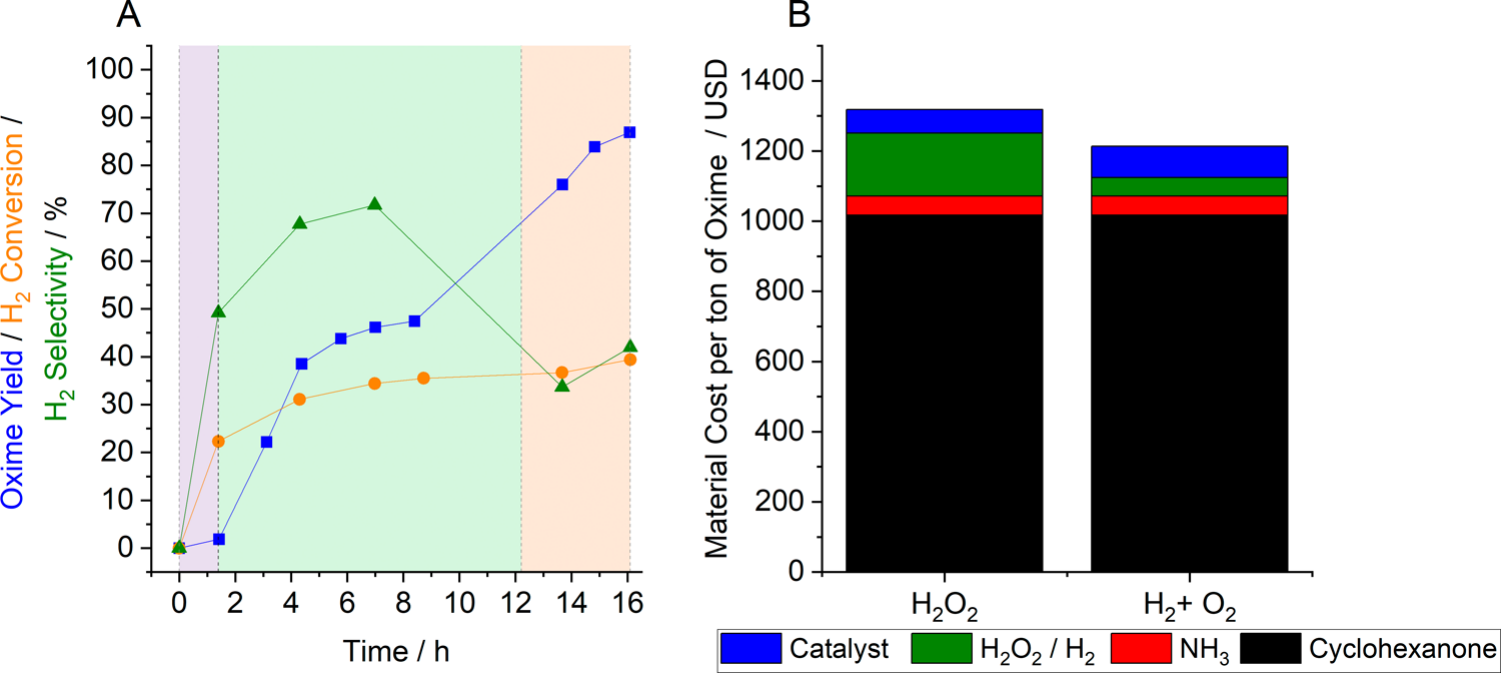
Industrial viability of the in situ approach to cyclohexanone ammoximation. (A) Cyclohexanone ammoximation via in situ H_2_O_2_ synthesis under industrially relevant conditions. (B) Techno-economic analysis comparison of the current commercial and in situ approaches to cyclohexanone ammoximation. Reaction conditions (A): Cyclohexanone (19 wt %): NH_3_ (aq) (1:1 (mol/mol)), 3.6% H_2_, 6.4% O_2_, 90% N_2_ (580 psi, 20 mL min^–1^), 0.33% Au 0.33% Pd/TS-1(acetate-O + R):Al_2_O_3_ (4:1) (catalyst mass = 0.42 g), *t*-BuOH: H_2_O (9:1 (v/v), 0.005–0.10 mL min^–1^), residence time 75 min at 0.01 mL min^–1^ liquid flow rate and 150 min at 0.005 mL min^–1^ liquid flow rate, reaction temperature 80 °C. Note 1: The catalyst was reduced (2 h, 200 °C, H_2_) following an initial oxidative heat treatment (16 h, 110 °C, static air). Note 2: Reaction conditions between 0 and 1.4 h: As above with liquid flow of 0.1 mL min^–1^ (purple background). Note 3: Reaction conditions between 1.4 and 12.3 h: As above with liquid flow of 0.01 mL min^–1^ (green background). Note 4: Reaction conditions between 12.3 and 16 h: As above with liquid flow of 0.005 mL min^–1^ (orange background). Note 5: The reader is directed to our original work for a complete techno-economic analysis.^4^

In the case of our low-loaded AuPd composite catalyst (0.33% Au-0.33% Pd/TS-1), no clear loss in catalyst stability was detected over 250 h on stream under industrially relevant conditions. Indeed, through-process optimization oxime yields approaching 90% may be obtained (Figure 3A). A subsequent techno-economic analysis revealed that, compared to the current state-of-the-art industrial process, considerable cost reductions (*ca*. 15%) may be achieved through the in situ approach (Figure 3B). Notably, such calculations are based on material cost alone and do not account for reduced handling and storage costs or attempt to quantify savings associated with reduced GHG emissions. While we have demonstrated the potential for such chemistry to rival the current industrial process and consider that such an approach may be applied to alternative transformations reliant on a combination of H_2_O_2_ and TS-1, it is important to note that evaluation at scale and over industrial time scales is still required.

## Alcohol Oxidation

**Figure 4:**
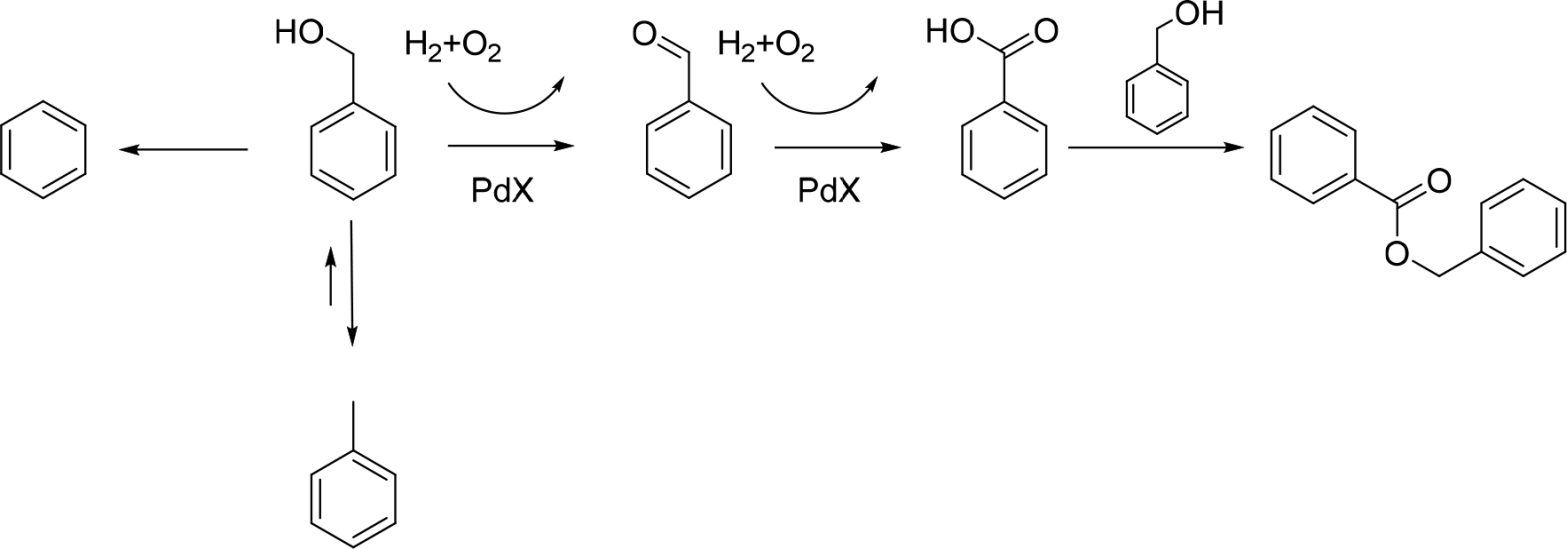
Proposed reaction scheme for the oxidation of benzyl alcohol via in situ H_2_O_2_ synthesis.

Alcohol oxidation utilizing molecular oxygen represents an environmentally friendly route to the synthesis of aldehydes.^44^ However, while the green credentials of O_2_-mediated valorization are evident, further improvements in process efficiency may be realized through the use of H_2_O_2_, which typically allows for significantly lower operating temperatures and may facilitate improved reaction selectivities. In particular, we have focused our attention on the selective oxidation of benzyl alcohol (Figure 4), with initial studies based on AuPd catalyst formulations^32,33^ which have been extensively studied for both H_2_O_2_ direct synthesis^45^ and aerobic alcohol oxidation.^44,46^

**Figure 5:**
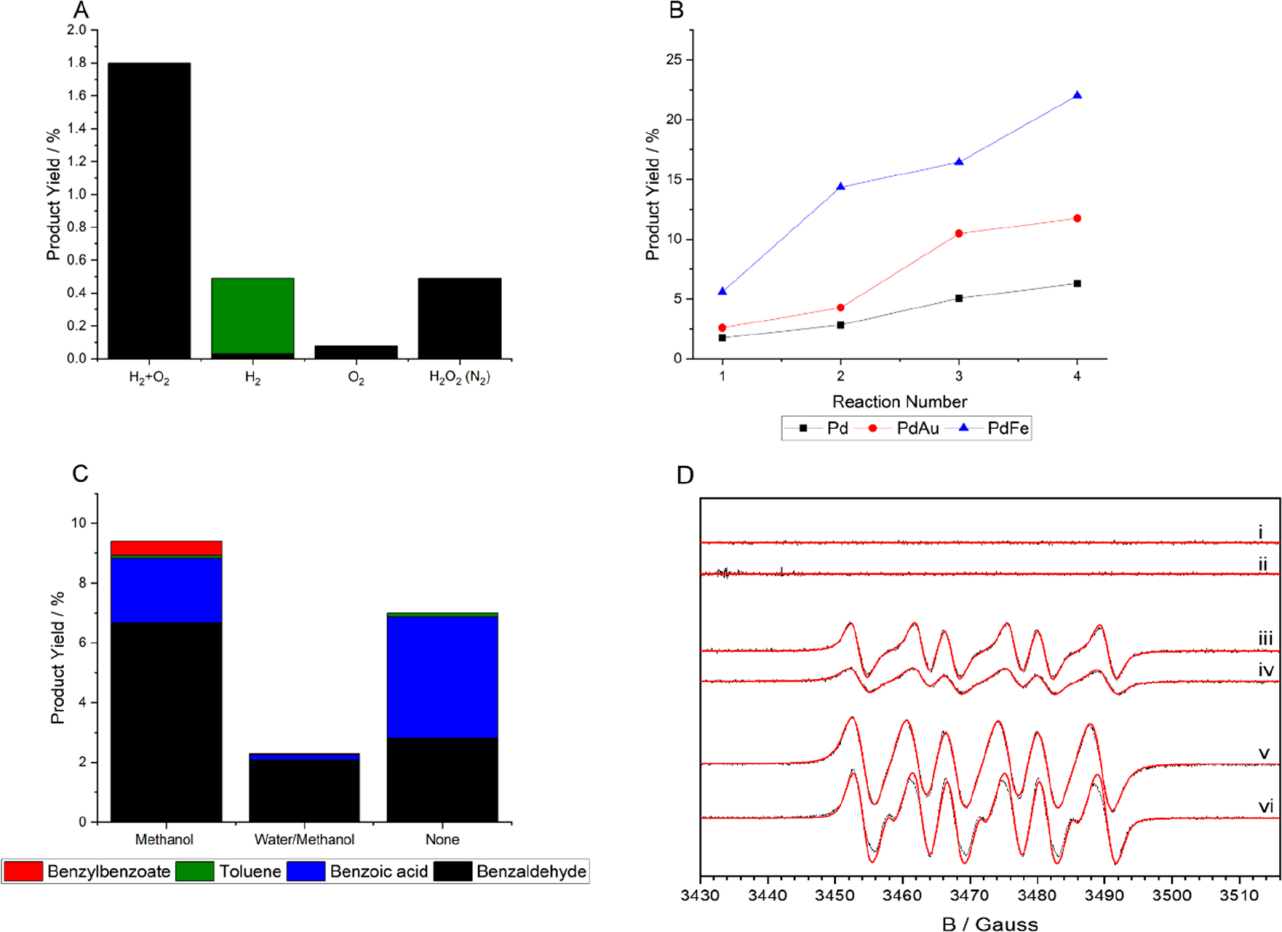
Benzyl alcohol oxidation via in situ H_2_O_2_ synthesis. (A) Comparative performance of in situ H_2_O_2_ synthesis toward the oxidation of benzyl alcohol using a 1% Pd/TiO_2_ catalyst. (B) Comparison of the catalytic activity of 1% Pd/TiO_2_, 1% PdAu/TiO_2_, and 1% PdFe/TiO_2_ catalysts over sequential reactions. (C) Effect of reaction solvent on the performance of a 5% AuPd/TiO_2_ catalyst. (D) Experimental (black) and simulated (red) EPR spectra of DMPO-radical adducts formed during the oxidation reaction. Reactions were conducted in the presence of DMPO and (i) 1% Pd/TiO_2_ (0.083 h), (ii) 1% Pd/TiO_2_ (0.5 h), (iii) 1% PdAu/TiO_2_ (0.083 h), (iv) 1% PdAu/TiO_2_ (0.5 h), (v) 1% PdFe/TiO_2_ (0.083 h), and (vi) 1% PdFe/TiO_2_ (0.5 h). Reaction conditions: Catalyst (0.01 g), benzyl alcohol (1.04 g, 9.62 mmol), solvent (7.1 g), 5% H_2_/CO_2_ (420 psi), 25% O_2_/CO_2_ (160 psi), 0.5 h, 50 °C, 1200 rpm. Note 1: In A, the concentration of commercial H_2_O_2_ used is comparable to that produced if all H_2_ in a standard in situ reaction is converted to H_2_O_2_. H_2_O_2_ was not continually introduced into the reactor. N_2_ in parentheses is indicative of a gaseous atmosphere (580 psi). For experiments carried out using H_2_ or O_2_ only, a gaseous mixture of 5%H_2_/CO_2_ (420 psi) or 25%O_2_/CO_2_ (160 psi) was used, with the total pressure being maintained at 580 psi using N_2_. Note 2: In A–D, the solvent used was MeOH.

The oxidation of benzyl alcohol is an often-used model transformation for alcohol upgrading due to the limited number of products and the well-understood mechanisms involved in their formation. Importantly, the primary product, benzaldehyde, also finds wide-scale applications in perfumery and in the agrochemical sector. The catalytic generation of oxygen-based radicals is known to be key in the oxidative mechanism, catalyzing proton abstraction from the alcohol moiety,^47^ with the presence of radical quenchers shown to effectively suppress catalytic performance.^33^ Notably, it is crucial to ensure a continual supply of H_2_O_2_ (and therefore oxygen-based radicals) in order to maintain high selectivity toward benzaldehyde. Indeed, when limited by H_2_O_2_ availability, Pd-catalyzed disproportionation pathways are favored, leading to the production of toluene,^33^ and as such, it is also crucial that competitive H_2_O_2_ degradation pathways are inhibited in order to achieve high process efficiency. Likewise, the continual supply of stabilizer-free H_2_O_2_ via an in situ approach avoids the dilution of product streams associated with the use of commercial H_2_O_2_ and the need for the separation of proprietary stabilizing agents while also offering high efficacy toward benzyl alcohol valorization compared to that offered by the preformed oxidant. Indeed, the in situ approach also greatly outperforms the purely aerobic pathway, which can be attributed to the requirement for relatively high operation temperatures when utilizing oxygen as the terminal oxidant (Figure 5A).

The electronic and structural modifications that result from the formation of AuPd nanoalloys are known to promote catalyst efficacy toward alcohol oxidation, and in part, this is achieved through the control of the Pd oxidation state, a key parameter given the presence of H_2_ and the enhanced selectivity of Pd^2+^ (i.e., PdO) species toward H_2_O_2_, compared to Pd^0^ analogues.^3^ However, it is likely that structural effects also contribute to the improved performance of alloyed species, compared to the Pd-only analogue.^48^ Our observation of relatively large concentrations of residual H_2_O_2_ in benzyl alcohol oxidation product streams,^32^ as a result of improved catalytic selectivity toward H_2_O_2_, combined with the general acceptance of radical-mediated pathways prompted us to explore catalyst formulations alternative to those centered around AuPd. In particular, motivated by concurrent studies into pollutant remediation, we have focused on the development of bifunctional catalysts that are able to synthesize H_2_O_2_ in situ and subsequently catalyze the production of oxygen-based radicals. The alloying of Pd with Fenton’s active metals, in particular Fe, was found to be highly effective, with optimal catalyst formulations achieving product yields double that of the AuPd analogue, with high selectivity toward benzaldehyde observed (Figure 5B).^3^

The presence of low concentrations of benzyl alcohol is well known to promote selectivity toward benzaldehyde and prevent overoxidation to benzoic acid.^49^ This is achieved by intercepting the benzoylperoxy radical species which catalyze competing reaction pathways, resulting in the formation of perbenzoic acid, and ultimately benzoic acid, via a non-Baeyer–Villiger-type oxidation process.^49^ Interestingly, the presence of an aliphatic alcohol solvent, in this case methanol, is also able to promote process efficiency, improving both the rate of benzyl alcohol conversion and selectivity toward the aldehyde (Figure 5C).^3,33^ While this may be attributed to the improved solubility of gaseous reagents, particularly H_2_, and a subsequent improvement in H_2_O_2_ synthesis rates, we have also observed the noninnocent nature of the solvent via electron paramagnetic resonance (EPR) spectroscopy (Figure 5.D).^3^ These studies revealed the presence of O-centered methoxy radicals (CH_3_O·), which result from the scavenging of hydroperoxyl and hydroxyl radicals by the solvent. Notably, the primary radical species (·OOH, ·OH, ·O_2_^–^), were observed only in the presence of AuPd and PdFe catalysts and were absent over the Pd-only analogue, suggesting a possible surface-mediated mechanism over the latter formulation, which aligns well with investigations into Pd and AuPd nanoparticles for H_2_O_2_ synthesis.^50^ As may have been expected based on product yields, the concentration of radical species was found to be significantly greater over the PdFe formulation compared to the AuPd analogue. However, the presence of low concentrations of residual H_2_O_2_ over the PdFe formulation suggests that further improvements may yet be achieved through further catalyst design, particularly if the in situ approach is to compete with the exceptional yields which may be achieved by high-temperature aerobic oxidation.

## Chemo-Enzymatic Cascades

The selective oxidative valorization of hydrocarbon C–H bonds represents a longstanding challenge for heterogeneous catalysis, with overoxidation and low regioselectivity typically inhibiting process efficiency and necessitating limited conversion rates in order to prevent the formation of undesirable byproducts. Alternatively, a number of H_2_O_2_-mediated enzymatic approaches have been developed, including those centered around chloroperoxidases (CPOs) and the closely related class of unspecific peroxygenases (UPOs). Although highly selective, such enzymatic approaches suffer from low sensitivity to even moderate concentrations of H_2_O_2_. In order to avoid these concerns and the continual dilution of product streams that would result from the utilization of commercial H_2_O_2_, numerous groups have sought to generate H_2_O_2_ in situ, with photo- and electro-catalytic methodologies reported. However, the scale-up of such approaches would be challenging, and concerns around enzyme lifetime remain. Accordingly, a number of coenzymatic systems have been developed to directly supply H_2_O_2_ to peroxygenases for C–H bond activation and include the use of glucose oxidase (GOx), formate oxidase (FOx), and choline oxidase (ChOx). However, such multienzymatic cascades have suffered from the need for continual pH maintenance, poor atom efficiency, and the formation of large quantities of byproducts, which represent a source of enzyme deactivation if not removed from the reactor system.

**Figure 6:**
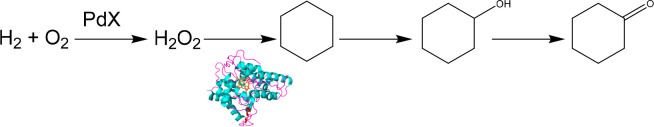
Proposed reaction pathways associated with the chemo-catalytic/enzymatic valorization of cyclohexane via in situ H_2_O_2_ synthesis.

Alternatively, we have recently developed a tandem chemo-catalytic/enzymatic approach centered around the direct synthesis of H_2_O_2_ over Pd-based heterogeneous catalysts coupled with the unspecific oxygenase PaDa-I (Figure 6), bridging the wide conditions gap that exists between the two processes and achieving product yields several orders of magnitude greater than that achieved over an in situ approach purely reliant on chemo-catalysis.^28,29^

In particular, we have demonstrated the efficacy of the chemo-catalytic/enzymatic system over a wide range of substrates, achieving total turnover numbers rivaling the most efficient approaches reported in the literature^1^ while also maintaining the high stereoselectivity one may expect of enzymatically mediated transformations (Table 1). Indeed, through control of nanoparticle composition, it is further possible to modulate reaction pathways, controlling H_2_O_2_-mediated enzymatic oxidation and chemo-catalyzed hydrogenation and, in the case of our exemplar study, allowing for the production of either primary or secondary alcohols from terminal alkenes.^51^

**Table 1:**
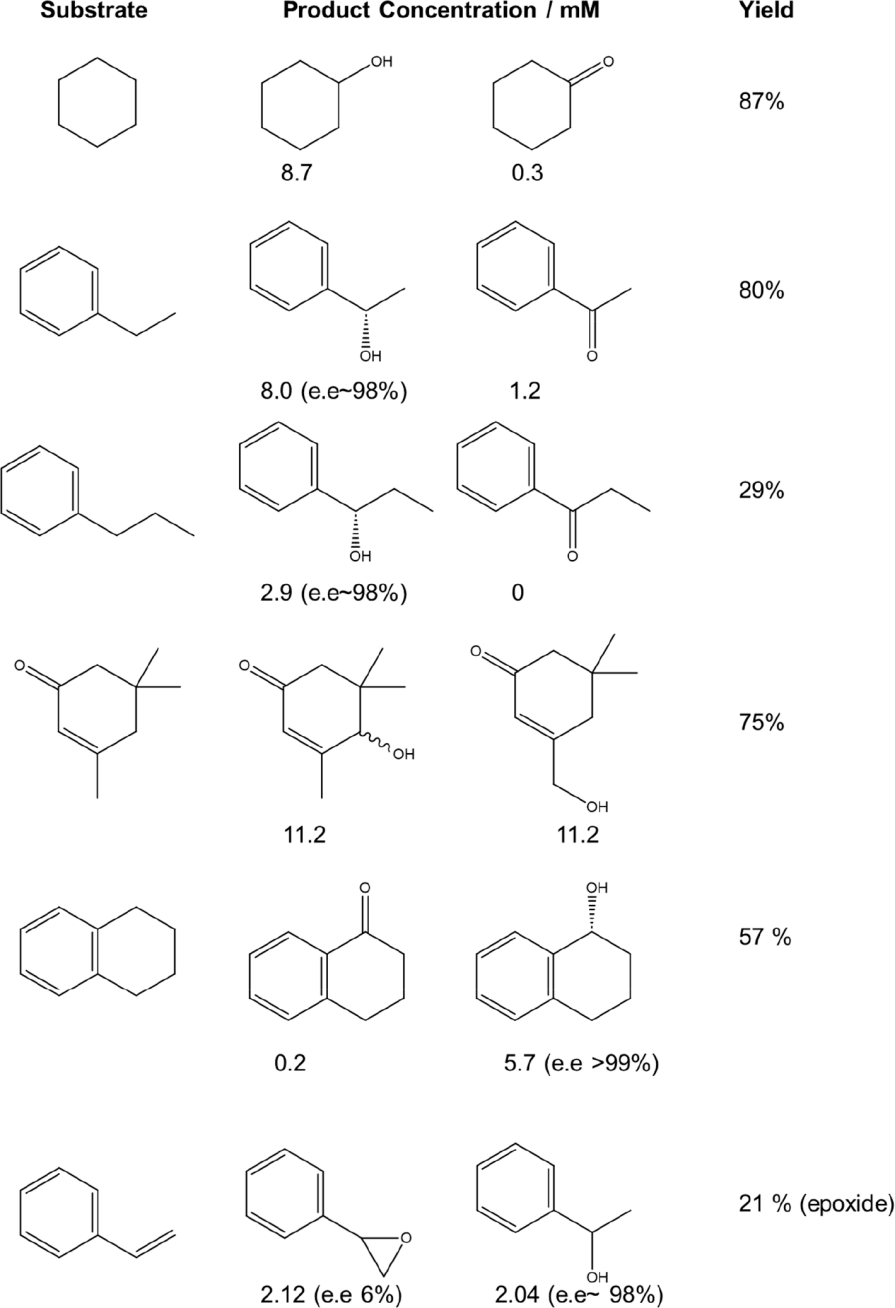
Substrate Scope of the Chemo-Enzymatic Cascade towards C–H Bond Hydroxylation^a^

a Reaction conditions: Catalyst (0.001 g), substrate (10 mM), PaDa-I (15 U mL_RM_^–1^), phosphate buffer (100 mM, 10 mL, pH 6.0), using a gas mixture of 80% H_2_/air H_2_ (23 psi) and air (6 psi), 2 h, 20 °C, 250 rpm. Note: The yield is reported as that of the primary hydroxylated product.

**Figure 7:**
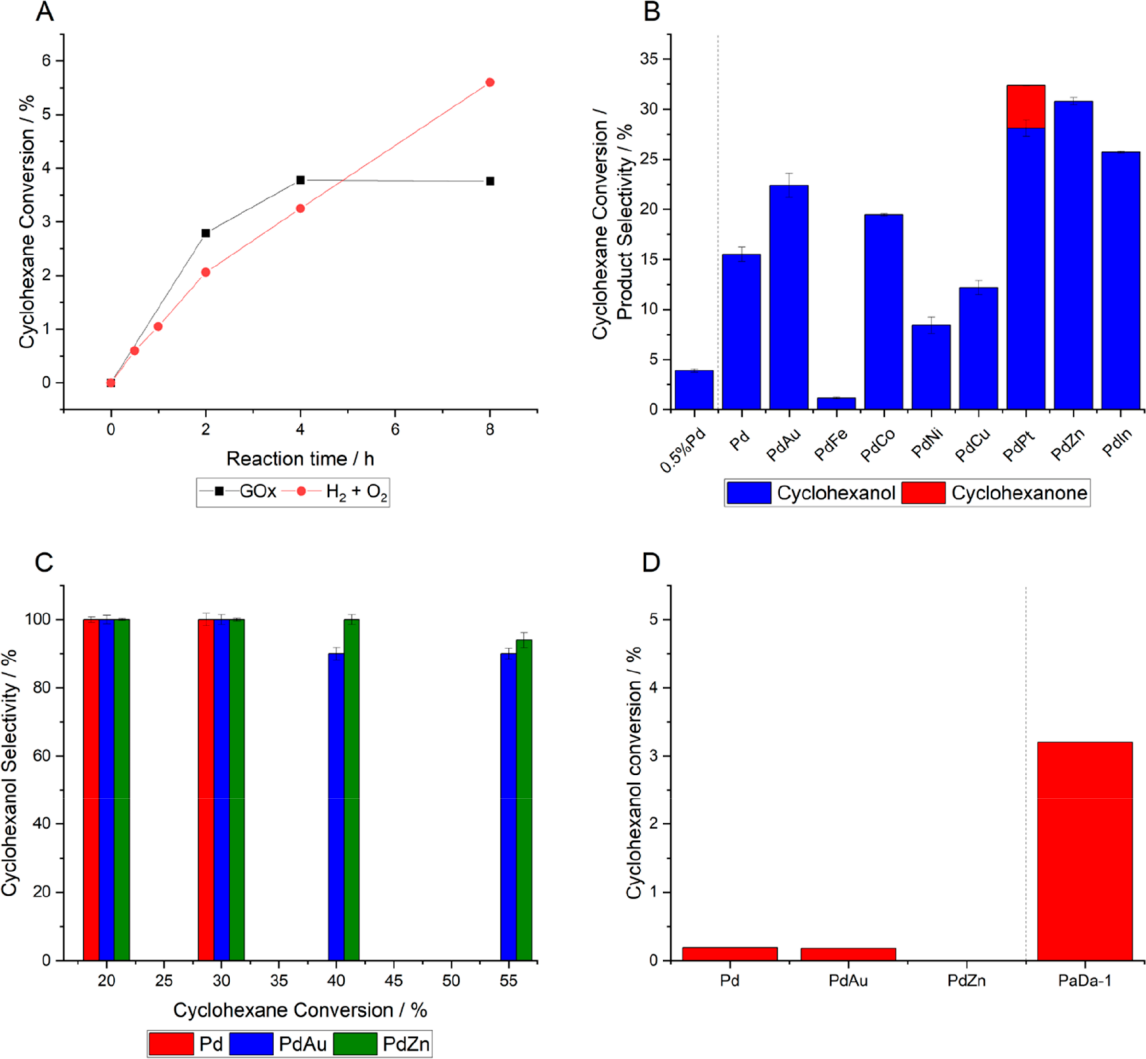
Chemo/enzymatic C–H bond oxidation, via in situ H_2_O_2_synthesis. (A) Comparison of the chemo-catalytic/enzymatic system (where H_2_O_2_ is synthesized over a 5% AuPd/TiO_2_ catalyst) with the coenzymatic approach using glucose oxidase (GOx, 0.2 UmL_RM_^–1^) and 200 mM glucose under an oxidative atmosphere (air, 6 psi). (B) Product selectivity as a function of catalyst formulation when used in conjunction with PaDa-I. (C) Chemo-catalytic and (D) enzymatic overoxidation of cyclohexanol. Reaction conditions: Catalyst (0.001 g), substrate (10 mM), PaDa-I (15 U mL_RM_^–1^), phosphate buffer (100 mM, 10 mL, pH 6.0), using a gas mixture of 80% H_2_/air H_2_ (23 psi) and air (6 psi), X h, 20 °C, 250 rpm. Note 1: In B–D, the catalyst formulation is 1% PdX/TiO_2_ (Pd:X = 1:1 (w/w)).

Although the combination of PaDa-I with AuPd chemo-catalyst formulations is highly effective for the hydroxylation of unactivated sp^3^ carbons, significantly outperforming the activity of coenzymatic systems (Figure 7A), further process efficiencies may be obtained through (1) the improved selective utilization of H_2_ (indeed, in the case of cyclohexane hydroxylation selectivity based on H_2_ in the AuPd/PaDa-I system is as low as 10%),^1^ (2) the inhibition of purely enzymatic and chemo-catalyzed over oxidation pathways (Figure 7B–D),^52^ and (3) improving H_2_O_2_ synthesis rates (Figure 7B)^53^ in order to ensure a continual supply of desirable concentrations of the oxidant.

However, it is important to note that due to the sensitivity of the UPO enzyme to H_2_O_2_ there is a requirement for the reaction to remain limited by H_2_O_2_ availability, rather than its subsequent utilization in oxidative valorization, to promote enzyme stability. Indeed, such concerns exist for all approaches which seek to continually supply H_2_O_2_ for enzymatic utilization.

While the potential of this technology is particularly exciting, given the high product selectivities and yields achieved, it is important to note that several hurdles must first be overcome if it is to rival industrially operated processes. From a safety perspective, it is imperative that explosive mixtures of H_2_/O_2_ are avoided. To date, our studies have utilized gaseous reagent mixtures above the upper explosive limit to ensure that the process is not limited by H_2_ availability. However, it would be beneficial to operate under H_2_-lean conditions (i.e., under the lower explosive limit), particularly if concerns around gaseous substrate availability can be minimized and indeed in doing so one may expect potential competitive hydrogenation pathways to be minimized. With this in mind, we consider recent reports by Hollmann and co-workers, which have demonstrated the improved stability of unspecific peroxygenases in the presence of a range of organic cosolvents to be particularly noteworthy, given the improved reagent solubility which may result from the use of such mixed-solvent systems.^54^ Despite the improvements in enzyme stability that can be obtained through modulation of the solvent composition, it is clear that the stability of the free enzyme, under large-scale operation, as well as issues with separation from product streams, would lead to additional complexity. The use of an immobilized enzyme would, in particular, overcome purification concerns and would allow for operation in a continuous/semicontinuous mode, and indeed enzyme immobilization has been reported to lead to improved lifetimes. As such, it is recommended that enzyme immobilization is treated as a priority in future studies, particularly with a focus on the development of composite materials that consist of the chemo-catalytic and enzymatic components.

**Figure 8:**
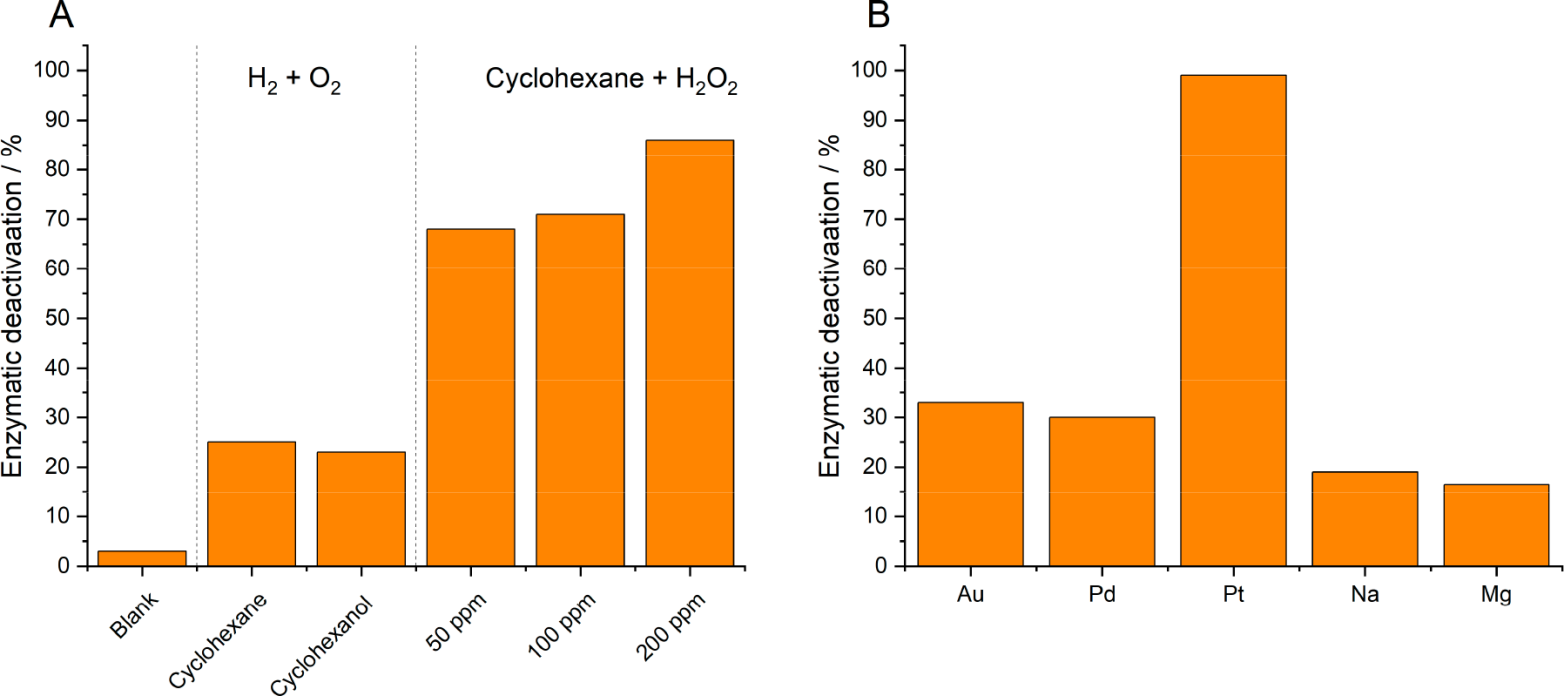
Sources of enzyme deactivation in the chemo-enzymatic approach to cyclohexane oxidation. (A) Effect of key reaction parameters and (B) homogeneous metal species on enzyme deactivation, as determined by ABTS assay. ABTS assay reaction conditions for (A) and (B): 100 μL of the reaction solution was added to 900 μL of ABTS solution (100 mM sodium citrate–phosphate buffer, pH 4.4 with 0.3 mM ABTS and 2 mM H_2_O_2_), and substrate conversion was followed by measuring the absorption at 418 nm (ε 418 = 36 000 M^–1^ cm^–1^) at 30 °C. Note for A: In the case of the blank experiment, the UPO was stirred under ambient conditions and the pressure of the buffer only. In the case of the cyclohexanol experiment, the concentration of cyclohexanol was equivalent to that present at 30% conversion of cyclohexane. The blank cyclohexane oxidation reaction conditions for (B): Metal salt ((5.1–9.4) × 10^–9^ M), cyclohexane (10 mM), PaDa (15 U mLRM^–1^), and phosphate buffer (100 mM, 10 mL, pH 6.0), using a gas mixture of 80% H_2_ (23 psi) in air (6 psi), 2 h, 250 rpm.

While H_2_O_2_ concentration is a major contributor to enzyme deactivation, we have also observed a degree of deactivation associated with the presence of both the organic substrate and major reaction products (in this case cyclohexane and cyclohexanol, respectively) (Figure 8A). Furthermore, while our chemo-catalytic formulations have proven to be stable over our chosen time frame (up to 8 h), we have identified the contribution of leached metal species (Au, Pd, and Pt), as well as those common ions typically found in water (Mg, Na, and Cl), to enzyme deactivation (Figure 8B). Such observations highlight the need for a holistic understanding of any chemo-enzymatic process, particularly given the relative costs associated with enzyme synthesis.^53^

## Water Treatment

The bioremediation of contaminated water bodies and degradation of low levels of bioactive compounds (such as exogenous hormones and pharmaceuticals) and organic pollutants released from agricultural, industrial, and urban activities into groundwater represents a growing challenge to aquatic and human health. These concerns are compounded by the limited degradation efficacy of conventional chemical treatments, such as chlorination, to recalcitrant remediation and the growing health concerns associated with the chemical residues that result from traditional disinfection agents.^55^ As such, growing interest has been placed on the application of advanced oxidation processes (AOPs) that utilize the high reactivity of oxygen-based radicals (·OH, ·OOH, and ·O_2_^–^) for water disinfection, with the combination of H_2_O_2_ and O-zone (H_2_O_2_/O_3_) or UV irradiation (H_2_O_2_/UV), well studied. However, while effective, the costs associated with the use of oxidizing reagents (H_2_O_2_ and O_3_) and practical limitations of high-energy light sources as well as the presence of H_2_O_2_ stabilizing agents will likely preclude their application at scale and have led our laboratory to investigate the generation of these reactive oxygen species in situ through the combination of H_2_ and O_2_.

**Figure 9:**
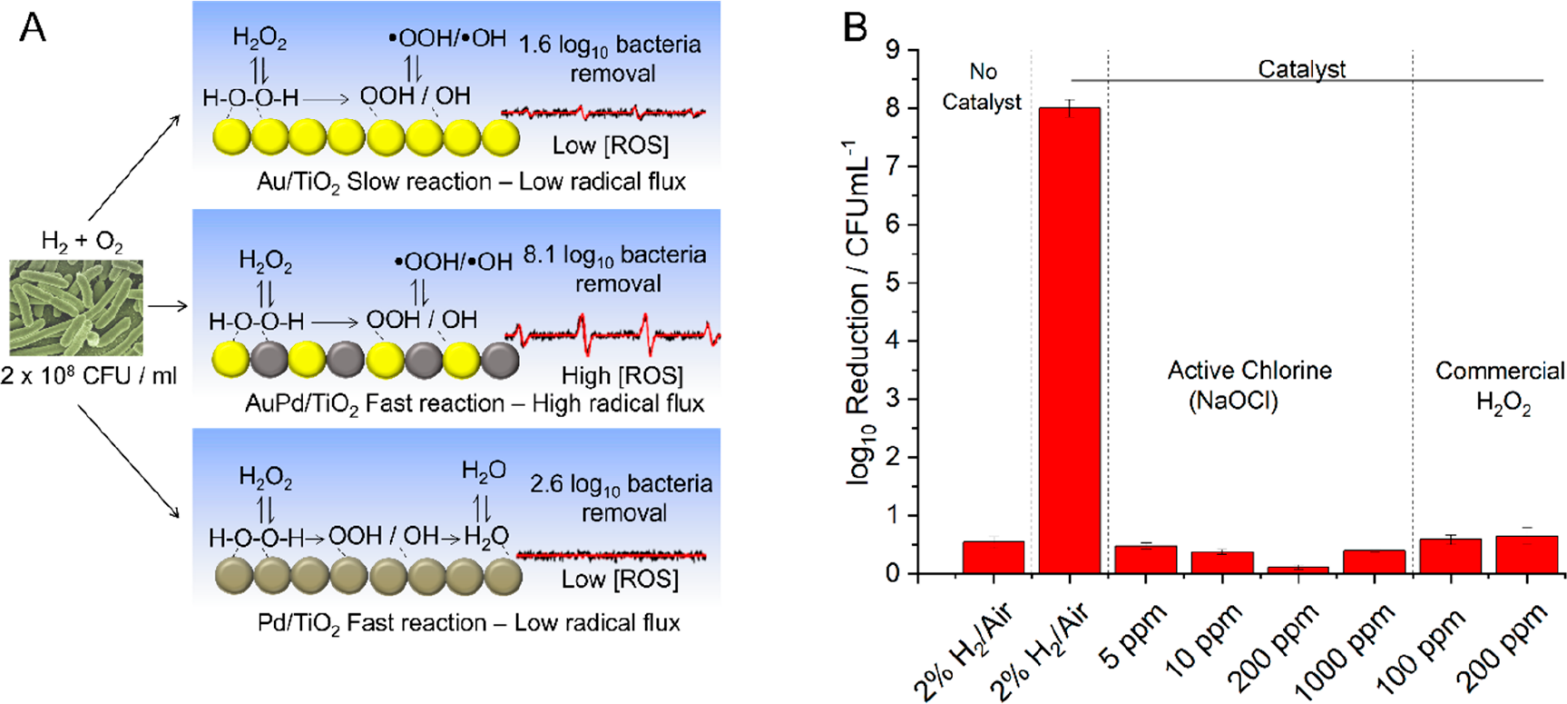
Bioremediation via in situ H_2_O_2_ synthesis. (A) Proposed reaction scheme for the in situ remediation of *E. coli*. K12 JM109 by reactive oxygen species generated over AuPd surfaces, summarizing our observations of catalytic performance and O-centered radical speciation. (B) Comparison of microbicidal activity using aqueous NaOCl, preformed H_2_O_2_, and in situ-synthesized H_2_O_2_. Reaction conditions: Catalyst (0.12 g), 2% H_2_, 20% O_2_, 78% N_2_, (145 psi, 42 mL min^–1^), reaction solution (H_2_O and *E. coli* K12 JM109 (2 × 10^8^ c.f.u. mL^–1)^ 0.2 mL min^–1^ liquid flow), 0.5 h, 2 °C.

Utilizing dilute streams of H_2_ (at levels comparable to those generated via water splitting) and air to synthesize stabilizer-free H_2_O_2_ and short-lived reactive oxygen species over AuPd nanoalloys, we have established that our chemo-catalytic approach can offer excellent bactericidal and virucidal efficacy.^2^ The presence of Au is crucial in the release of intermediate oxygen-based species from catalytic surfaces, as identified through extensive EPR spectroscopy (Figure 9A). Indeed, our technology achieves an 8.1 log_10_ reduction in *E. coli* levels (Figure 9B) and an 8.0 log_10_ reduction in the viability of the nonenveloped virus MS2, a surrogate for the polio virus, representing a 99.999999% reduction in pathogen viability after a contact time of 30 s, which is several orders of magnitude more effective than that achieved through the use of preformed H_2_O_2_ or through chlorination, with these alternative approaches requiring relatively long contact times to offer appreciable levels of efficacy. Notably, in addition to oxygen-based radicals, low concentrations of residual H_2_O_2_, which offers broad spectrum bactericidal efficacy,^2^ are also synthesized, which may prolong the potable lifetime of the treated water and have been shown to aid in the prevention of biofilm formation, which represents a crucial challenge to water decontamination, again largely due to the poor efficacy of chlorination technologies.^2^

Alternatively, if combined with Fenton-active species, this residual H_2_O_2_ may act as a secondary source of oxygen-based radicals, in a manner similar to that observed for alkane^56−58^ and alcohol valorization.^3,33^ Inspired by these studies into radical-based selective oxidation, we subsequently turned our attention to the oxidative degradation of chemical pollutants. As with alcohol oxidation, the combination of Pd with Fe was found to be highly effective compared to monometallic Pd analogues or bimetallic formulations consisting of Pd with alternative Fenton-active metals (Cu, Mn, and Co) or Au.^59,60^ The poor performance of PdCu formulations in particular may be surprising, given the high efficacy of Cu-based catalysts in the oxidative valorization of alkanes^61^ when used in conjunction with preformed H_2_O_2_. However, it is important to note that theoretical studies have revealed the thermodynamic instability of intermediate hydroperoxyl species and in turn H_2_O_2_ over Cu-containing catalytic surfaces,^62^ with these studies aligning well with our experimental observations.^63,64^ Interestingly, we have recently established that the incorporation of dopant concentrations of Cu (<0.2% of total metal loading) into AuPd nanoalloys results in a considerable improvement in catalytic performance toward H_2_O_2_ direct synthesis, with these formulations offering comparable activities to those of better-studied AuPdPt systems.^65^ As such, these formulations may be considered to be attractive candidates for future use in water remediation or other in situ processes.^66,67^

**Figure 10:**
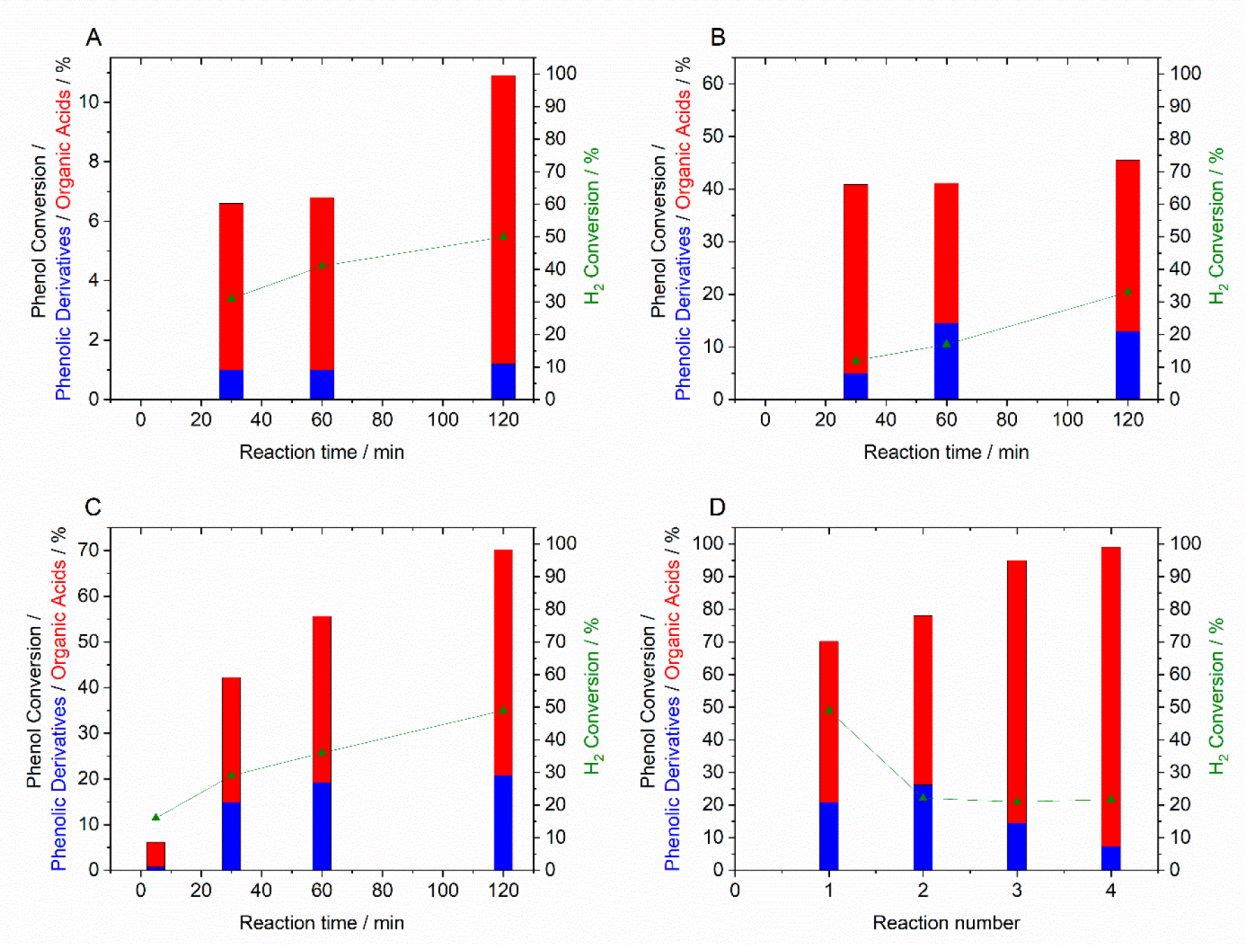
Oxidative degradation of phenol via in situ H_2_O_2_ synthesis. Comparison of catalytic activity using Pd-based catalysts (A) 1% Pd/TiO_2_, (B) 0.25% Pd-0.27% Fe/TiO_2_, and (C) 0.5% Pd/3% Fe-ZSM-5. (D) Performance of the 0.5% Pd/3% Fe-ZSM-5 catalyst over sequential reactions. Reaction conditions: Catalyst (0.01 g), phenol (1000 ppm, 8.5 g), 5% H_2_/CO_2_ (420 psi), 25% O_2_/CO_2_ (160 psi), 2 h, 30 °C, 2 h.

Clearly for any approach to chemical pollutant degradation to be effective there is a need to drive product distribution down the oxidative pathway, toward H_2_O and CO_2_. To date, our studies have focused on the total oxidation of phenol, an often-used proxy for pharmaceuticals and agrochemicals. In particular, phenol degradation represents a major challenge given the increased toxicity of partial oxidation products (catechol and benzoquinone and a range of diacids) and the propensity for such species to promote the leaching of catalytically active species, with organic acids in particular known to be strong chelating agents. Early catalyst formulations, prepared by wet coimpregnation techniques and utilizing common oxide supports (TiO_2_ and SiO_2_), were found to be somewhat effective (Figure 10A,B) but were unable to fully oxidize the phenolic derivatives while also suffering from considerable leaching of the Fe component. While the loss of active metal species is a concern for catalyst stability, it is important to note that homogeneous contributions toward the observed catalysis were found to be negligible. Subsequent studies, again focusing on PdFe formulations, demonstrated that considerable improvements in both activity and stability may be obtained through the incorporation of Fe into the zeolitic framework of ZSM-5 (Figure 10C,D).^68^

**Figure 11:**
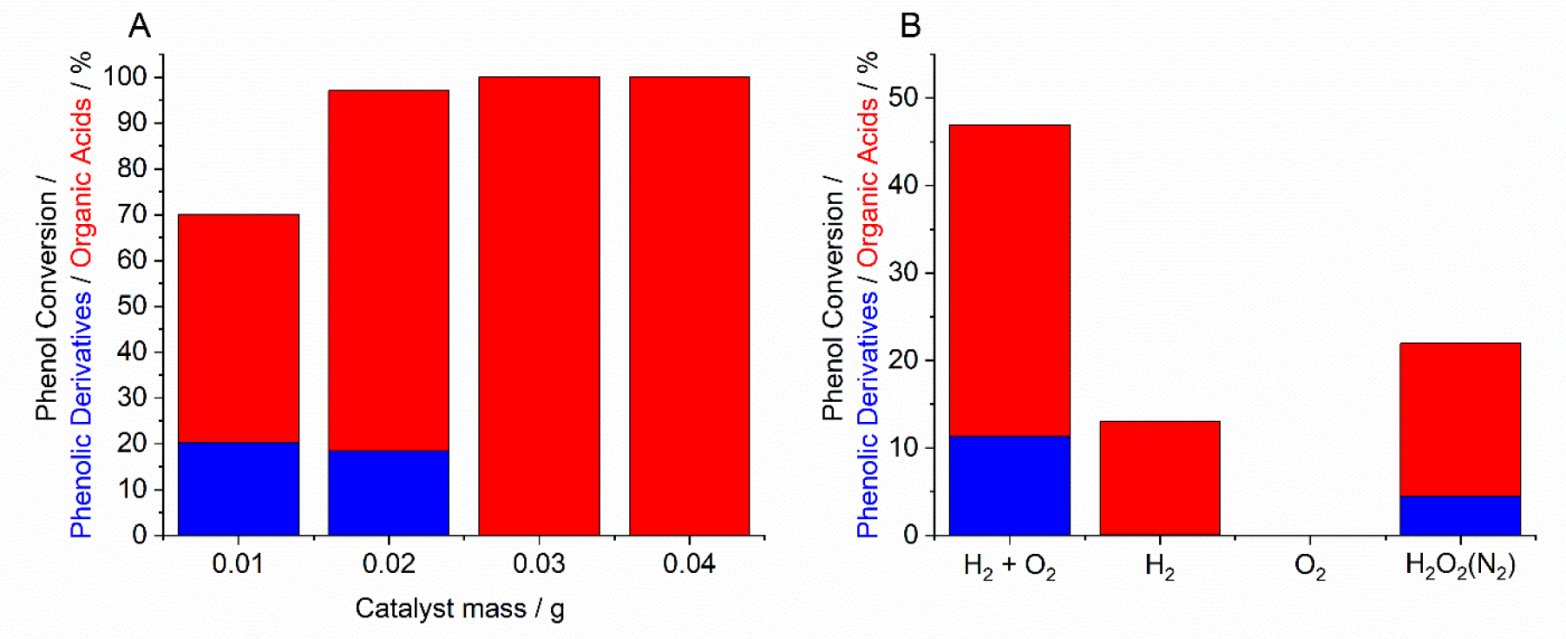
(A) Efficacy of the 0.5% Pd/3% Fe-ZSM-5 catalyst toward the oxidative degradation of phenol via in situ H_2_O_2_ synthesis as a function of the catalyst:phenol ratio. (B) Comparative performance of in situ H_2_O_2_ synthesis toward phenol degradation over a 0.5% Pd/0.5% Fe-ZSM-5 catalyst (0.0 1 g). Reaction conditions: Catalyst (X g), phenol (1000 ppm, 8.5 g), 5% H_2_/CO_2_ (420 psi), 25% O_2_/CO_2_ (160 psi), 2 h, 30 °C, 2 h. Note: In B, the concentration of commercial H_2_O_2_ used is comparable to that produced if all H_2_ in a standard in situ reaction is converted to H_2_O_2_. H_2_O_2_ was not continually introduced into the reactor. N_2_ in parentheses is indicative of the gaseous atmosphere (580 psi). For experiments carried out using H_2_ or O_2_ only, a gaseous mixture of 5% H_2_/CO_2_ (420 psi) or 25% O_2_/CO_2_ (160 psi) was used, with the balance consisting of N_2_.

Importantly, through the manipulation of the catalyst:substrate ratio, the optimal Pd/Fe-ZSM-5 formulation was also able to offer the total degradation of phenol, with complete selectivity toward the highly oxidized organic acids, representing a significant development compared to previous catalyst iterations (Figure 11A).^68^ While such technology is extremely promising, offering considerable improvements over the use of commercial H_2_O_2_ (Figure 11B) and demonstrating the potential role of catalysis in water treatment, further improvements in process efficacy are still required. With extended contact times currently required to achieve reasonable rates of phenol degradation, a focus should be placed on improving catalytic activity, particularly given the likely requirement for flow, rather than batch regimes to be utilized at scale. Additionally, there is a need to ensure high catalytic performance against a broad range of chemical pollutants, microorganisms, and a combination thereof, particularly using real-world water solutions. However, results to date are promising, and when used in combination with other emerging technologies, including biofiltration, we consider that the application of in situ-generated H_2_O_2_ and associated radical species may play a key role in safeguarding clean drinking water.

## Conclusions and Future Perspectives

Minimizing the use of finite resources and preventing the formation of pollutants must be a major focus if industrial chemical production is to be fit for the 21st century. Although still in its infancy, the effective generation and utilization of H_2_O_2_ (and its intermediates) in situ represents a potential sea-change in oxidative chemistry, allowing for considerable process intensification and moving the chemical synthesis sector closer toward a clean growth strategy.

Clearly, for many tandem chemical transformations that utilize H_2_O_2_, it is imperative that the rate of oxidant synthesis does not exceed the capacity of the secondary component to utilize the active species in selective oxidation, while in the case of radical-mediated transformations, there is a need to ensure effective utilization of the radical flux and minimize competitive termination reactions. Such transformations may be promoted through the careful selection of both catalyst formulation and reaction solvents, which can aid in radical propagation. There is an additional concern associated with the unselective utilization of H_2_, primarily through H_2_O_2_ decomposition but also via competitive substrate hydrogenation pathways, ensuring that high H_2_ selectivity is a major hurdle which must be overcome in order to achieve high process efficiency. While such considerations may direct future attention toward the development of catalyst formulations that are highly selective toward H_2_O_2_, it is also important to consider that in many cases there is rapid capture and utilization of H_2_O_2_ by the secondary active site. As such, we recommend that future studies focus on the development of catalyst formulations that are highly active toward H_2_O_2_ synthesis, with the caveat that the H_2_O_2_ production rate does not exceed that of its subsequent utilization.

We consider that the contributions to the field from our laboratory reported within this Account may offer considerable benefits to the chemical synthesis sector and beyond. However, in order to truly capitalize on such technology there is a clear need to progress beyond research-scale catalysts and toward technical-grade materials. Doing so will require a multidisciplinary collaborative effort involving specialists in characterization and material synthesis, in addition to theoreticians and chemical engineers, as well as necessitating improved dialogue between industry and academia and balancing the often competing interests and demands of these partners. It is only in adopting such a unified approach that we foresee development beyond the realm of academia and the true realization of the potential of this chemistry.
